# Factors influencing intentional non-utilization of healthcare: a study using the Andersen model

**DOI:** 10.3389/fpubh.2025.1503601

**Published:** 2025-04-09

**Authors:** Huanhuan Jia, Chunxia Miao, Xiaokang Song, Tianyu Feng, Yun Zhao

**Affiliations:** ^1^School of Management, Xuzhou Medical University, Xuzhou, Jiangsu, China; ^2^College of Public Health, Chongqing Medical University, Chongqing, China

**Keywords:** medical services, Andersen Healthcare Utilization Model, national survey, influencing factors, structural equation model

## Abstract

**Objective:**

This study aims to investigate the factors influencing residents' healthcare utilization behavior and provide a scientific basis for enhancing the overall efficiency of healthcare utilization.

**Methods:**

A comprehensive analysis was conducted using data from the China General Social Survey (CGSS) project. Exploratory Factor Analysis (EFA) and Structural Equation Modeling (SEM) were utilized to examine the influences and interrelationships of the three core factors of the Andersen Healthcare Utilization Model (Predisposing Factors, Enabling Resources, and Need), as well as the two extended factors (health behaviors and Medical-service Experience), on residents' decisions regarding the utilization of healthcare services.

**Results:**

A total of 2,230 participants were enrolled in this study. Most were male (55.74%), were married (85.38%), and had junior- and senior-high school educations (45.29%). Mean age was 52.39 years, and 56.32% of participants reported an annual income of <30,000 RMB. EFA distilled influencing factors into four domains: Predisposing and Enabling, Need, Health Behaviors, and Medical-service Experience. The results of the revised SEM indicated that the influence coefficients of Predisposing and Enabling, Need, and Medical-service Experience on Decision to Utilize Health Services (DUHS) were 0.095, −0.104, and 0.093 respectively. Mediation effect test results demonstrated that the indirect effects of Predisposing and Enabling, Need, and Health Behaviors on DUHS were −0.098, 0.024, and −0.017, respectively, all of which were statistically significant. Finally, the fit indices of the modified model indicated an acceptable model fit.

**Conclusion:**

This study showed that unmarried individuals with lower income and job instability exhibit reduced healthcare utilization due to economic barriers and lack of social support. Furthermore, medical service experience is another crucial factor affecting health service utilization. Notably, our findings suggest the need for targeted interventions, including enhanced insurance coverage, improving the quality of medical services and health education campaigns to mitigate disparities in access to health services.

## 1 Introduction

Due to the fast evolution of society and the exacerbation of population aging, residents' demand for health services has shown a pronounced upward trajectory (1, 2). Healthcare utilization, a pivotal metric gauging the efficacy and responsiveness of a nation's or region's healthcare system, has emerged as a paramount concern. It is particularly salient in China, a populous nation grappling with multifaceted challenges that include a rapidly aging society, profound urban vs. rural disparities in access to healthcare services, and an unequal distribution of medical resources (3, 4). These issues not only significantly affect the health and wellbeing of China's citizens but also pose direct threats to the sustainability of its healthcare system. Therefore, conducting exhaustive research into the patterns and determinants of residents' healthcare utilization behavior is of paramount urgency.

**Figure 1:**
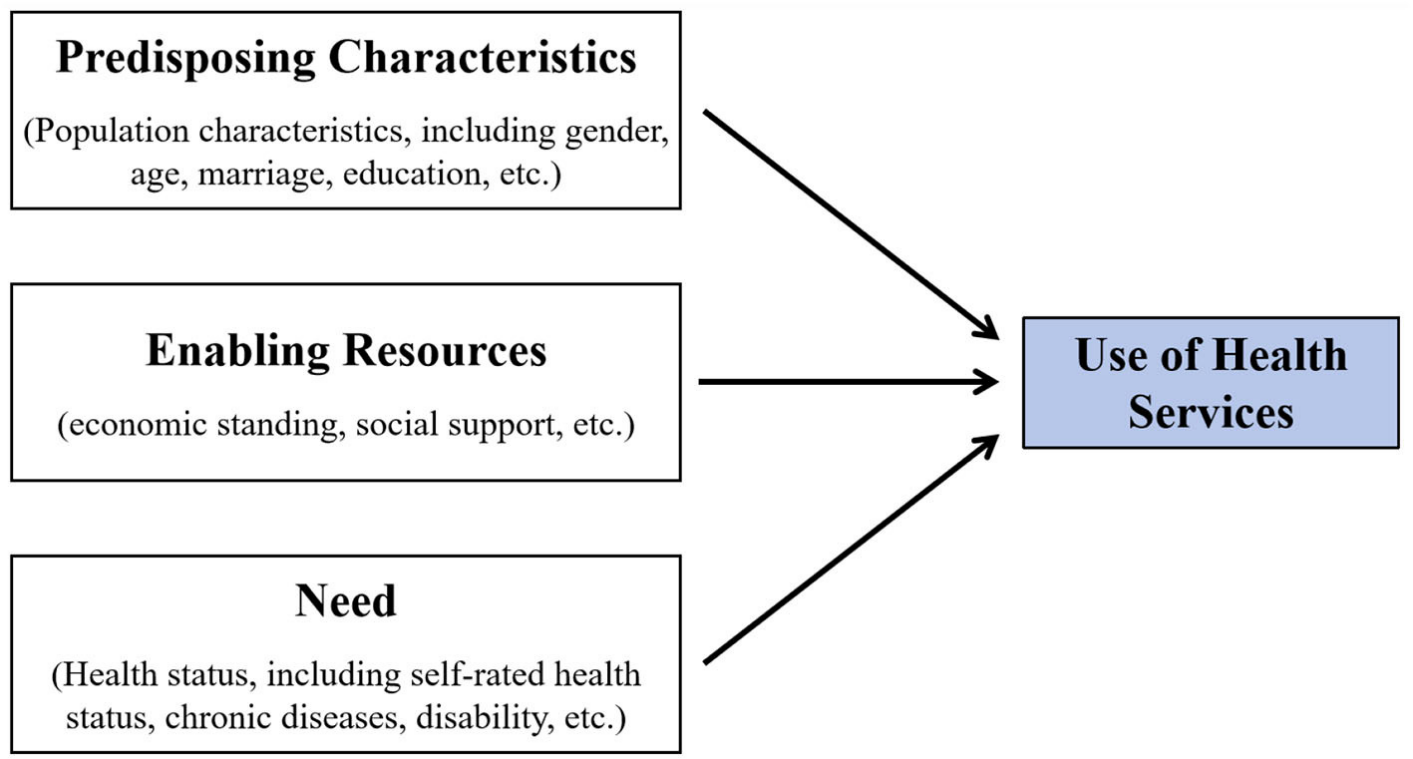
The conceptualization of Andersen Healthcare Utilization Model.

Utilization of healthcare by residents is a dynamic process driven by multiple intricate factors that span individual characteristics, the policy environment, the organizational structure of the healthcare system, and sociocultural contexts (5). To accurately discover the underlying mechanisms of this process and effectively inform policy formulation and practice enhancement, developing a comprehensive, systematic, and scientifically rigorous theoretical framework is imperative. To this end, various conceptual models have been developed and employed to explain and delineate the interrelationships among a series of possible predictive factors for healthcare utilization behavior, and to guide the analysis and evaluation of the study (6–8), including accessibility, availability, acceptability, affordability, adequacy, and the appropriateness of the final decision. Among them, the Andersen Healthcare Utilization Model is one of the most classic and recognized models. The Andersen Healthcare Utilization Model was initially proposed by the eminent American sociologist Ronald Andersen in 1968 (9). It is a multi-layered model that combines the individual and environmental determinants of health service use and has been most widely accepted and adopted in many countries (9–12), with its conclusions being convincing. It now stands as an indispensable, pivotal theoretical foundation for examining healthcare utilization behaviors. While various iterations and extensions of the model have emerged, they consistently emphasize the critical roles of three types of core factors influencing healthcare utilization: Propensity Characteristics (including fundamental demographic attributes such as gender and age), Enabling Resources (*e.g*., economic standing, social support), and Need (pertaining to an individual's state of health and specific manifestations of service requirements) (13–15). This conceptualization is visualized in Figure 1.

At the same time, there are slight differences in the research on the relationship between the three factors and the strength of their impact on healthcare utilization. Portuguese research has shown that needs and propensity Characteristics determine the use of health services by the older adult (16). A German study also indicates that needs are the main predictors of the cost of health insurance for the older adult (17). For specific populations, research in Australia and Turkey has also demonstrated that needs are the strongest predictors of mental health service use by depressed patients and of inpatients' readmissions (18, 19). Hence, among the three core factors, needs are an important predictor of the use of medical services. Moreover, the effects of the three factors on healthcare utilization vary among ethnic groups and regions (20). In China, through the Andersen Healthcare Utilization Model, researchers have a preliminary understanding of the influencing factors of healthcare utilization in China. It is pointed out that enabling resources can have a greater impact on healthcare utilization than predisposing factors and needs (21). However, a study has also pointed out that needs are the main predictors of rural residents' health service utilization in Guangxi, China (22). As can be seen, the impact of the three factors in the model on healthcare utilization varies by population and region. Additionally, a study has identified that predisposing characteristics, enabling resources, and needs have a local and neighborhood impact, which contributes to disparities in local health services utilization (23). Furthermore, researchers have also included variables such as social capital (24), service utilization experience (25), and social support (26) in the Andersen Healthcare Utilization Model according to specific research purposes to explore the impact of healthcare services utilization. In summary, extensive research on the Andersen Healthcare Utilization Model has been conducted, but the model structure and its results vary across different regions, populations, and research purposes.

The term “Non-utilization” has been referred to in prior researches. It implies that despite the availability of health services, people, due to various reasons such as economic factors, cognitive status, and social support, fail to access these services (22–24). Simultaneously, compared with other health service utilization models, the Anderson healthcare utilization model highlights the impact of individual factors on healthcare utilization, meaning it does not encompass the influence of health service providers. Therefore, this article employed a novel concept, “Intentional Non-utilization”. It indicates that the health service provider has offered the service without a lack thereof, and that when an individual is economically disadvantaged (with inadequate enabling resources) or has a cognitive bias regarding their own health condition (needs), there might be instances of deliberately not utilizing the already provided health-care services.

Therefore, with the Andersen Healthcare Utilization Model as a theoretical basis, this study employs “Intentional Non-utilization” to undertake a comprehensive analysis and systematic decomposition of the multiple factors influencing residents' healthcare utilization from the demand-side perspective. Additionally, a detailed examination of the complex interrelationships among these factors was planned to reveal the patterns governing residents' healthcare utilization behaviors, thereby providing a scientific basis for optimizing healthcare policies, enhancing the healthcare service system, and improving the overall efficiency of healthcare utilization.

## 2 Materials and methods

### 2.1 Data resources

The data used in this study were sourced from the well-regarded Chinese General Social Survey (CGSS), conducted by the Chinese Survey and Data Center at Renmin University of China (Beijing, China). Initiated in 2003, the CGSS stands as one of China's pioneering nationwide, comprehensive, and continuous academic surveys and is renowned for its authoritative results. The CGSS encompasses all urban and rural households across China's 31 provinces, autonomous regions, and municipalities. The sampling design employs a stratified three-stage probability sampling method, consisting of a mandatory stratum and a selected stratum. The mandatory stratum includes households from the urban districts of leading metropolitan cities, while the selected stratum covers other households nationwide. By employing scientific stratification and sample weighting, a sample of 12,000 households was extracted, maintaining an urban-to-rural ratio of 6:4. For detailed sampling procedures, please refer to the official website of the Chinese General Social Survey (http://cgss.ruc.edu.cn/index.htm). In 2021, the CGSS project systematically and comprehensively gathered data spanning multiple levels of Chinese society, encompassing communities, families, and individuals. This rich dataset, which includes age, gender, income, education, health behaviors, and healthcare utilization, provided a foundation for the detailed analysis in this study.

### 2.2 Selection and measurement of variables

Participants were queried about their actual past experience of whether they had elected not to utilize a resource or service in a specific situation. Therefore, we chose the question “*Do you intentionally avoid seeking medical attention when sick or injured?”* as the explanatory variable to understand their decisions and ascertain whether there is “Intentional non-utilization”. The answers were coded as NO (code = 1) and YES (code = 2). Furthermore, we identified five core variables by which to examine factors that influenced residents' healthcare utilization: (1) Predisposing Factors, (2) Enabling Resources, (3) Need, (4) Health Behaviors, and (5) Medical-service Experience.

#### 2.2.1 Predisposing factors

A systematic review of literature that employs Andersen's model revealed the six predisposing variables that are most frequently examined: age, marital status, gender, education, work, and region (12, 27). We incorporated these variables into our research. Age was treated as a continuous variable, while gender, marital status, and region were coded as dichotomous variables: Female (code = 1) vs. Male (code = 2), Unmarried (code = 1) vs. Married (code = 2), and Urban (code = 1) vs. Rural (code = 2), respectively. Respondents' educational levels were categorized in accordance with the 2011 International Standard Classification of Education (ISCED-2011) as follows: Primary education (ISCED 0–2) = 1, including primary and pre-primary education; Middle education (ISCED 3–4) = 2, including junior- and senior-high school education; and Higher education (ISCED 5–8) = 3, including undergraduate, master's, and doctoral studies. We also defined the following work status categories: unstable employment (code = 1), Self-employed (code = 2), and Employed (code = 3).

#### 2.2.2 Enabling resources

In previous research, income or financial status has been the most commonly employed variable in Enabling Resources (28). In addition, social support, defined as the support and assistance individuals receive from their social networks, has been identified as a crucial enabling resource influencing healthcare utilization, as shown by relevant studies on the application and refinement of Andersen's model (29, 30). In the Chinese context, social medical insurance represents a pivotal social-security system established through national and social legislation that aims to reduce the medical burden of insured persons and improve the level of medical security of the people, thereby mitigating the risk of poverty due to illness. Prior research has highlighted the significance of utilizing both public and commercial medical insurance as an economic factor that notably affects residents' utilization of medical services (31). Moreover, medical insurance has consistently emerged as an important predictor in studies using the Andersen model (15, 32). Therefore, in this study we designated Income, Social Support, and Medical Insurance as the core variables representing Enabling Resources. Specifically, Income was quantified using respondents' annual incomes; Social Support was assessed by asking whether respondents had someone to listen to their personal concerns regularly in the past year; and Medical-insurance status was categorized into four groups: No Medical Insurance (code = 1), Commercial Medical Insurance (code = 2), Social Security (code = 3), and Both Social Security and Commercial Insurance (code = 4).

#### 2.2.3 Need

Need pertains to an individual's requirement for healthcare, rooted in their specific state of health, which constitutes the most direct impetus for seeking such care. Currently, the Medical Outcomes Study 36-item Short-form Health Survey (SF-36) is the globally pre-eminent instrument for assessing quality of life (QoL), with its applicability validated across diverse populations (33, 34). The 8-item Short-form Health Survey (SF-8), a streamlined version of the SF-36 (35), covers eight domains essential to evaluating QoL over the preceding 4 weeks: general health (GH), physical functioning (PF), role limitations due to physical health problems (RP), bodily pain (BP), vitality (VT), social functioning (SF), mental health (MH), and role limitations due to emotional problems (RE). The SF-8 scale has been widely adopted in international research due to its brevity, ease of administration, and high response rates (36). Therefore, in this study we used the SF-8 to gauge participants' health characteristics, thereby mirroring their healthcare needs.

#### 2.2.4 Health behaviors

In their assessments of healthcare utilization, scholars have found that in addition to the three core factors posited in the Anderson model, personal-health behaviors strongly correlate with health education or awareness, emerging as a pivotal influence on healthcare demand and service utilization (37, 38). Consequently, in this study we integrated Health Behaviors into the Anderson model. Our review of pertinent literature on Health Behaviors led us to select three representative factors as core variables. First, the detrimental effects of smoking on health and its nexus with healthcare utilization have been amply documented (39, 40); therefore, we deemed respondents' smoking status a crucial health behavior and coded it as Smoking (code = 1) vs. Non-Smoking (code = 2). Second, exercise mitigates the risks of cardiovascular diseases, type 2 diabetes, certain cancers, depression, and anxiety (41); conversely, physical inactivity heightens vulnerability to various health challenges (42), ultimately increasing healthcare utilization (43). Therefore, we gathered data on the frequency of leisure time physical exercise among participants, categorizing them as Never (code = 1), Several Times a Year or Less (code = 2), Several Times a Month (code = 3), Several Times a Week (code = 4), and Every Day (code = 5). Third, undergoing physical examinations demonstrates an individual's concern for their health, constituting a vital Health Behavior. Research indicates that individuals with higher rates of physical-examination utilization tend to adopt healthier lifestyles and behaviors (44); moreover, this population reports better self-assessed state of health (45), and regular physical examinations correlate negatively with healthcare utilization (46). Therefore, we collected data on the frequency of physical examinations sought out by participants in the past 3 years, classifying them as Never (code = 1), Irregular Physical Examination (code = 2), and Regular Physical Examination (code = 3).

#### 2.2.5 Medical-service experience

Due to the high professional standards of healthcare services and the profound lack of medical knowledge among patients, medical personnel often occupy a dominant position in the delivery of medical care, significantly influencing patient experience and satisfaction. Extensive research has emphasized the crucial role of effective communication between patients and medical staff in fostering high satisfaction levels (47). First, positive demeanor, professional commitment, and competence of medical staff have been found to correlate positively with patient satisfaction (48, 49). Second, patients tend to report heightened satisfaction when they receive ample assistance throughout the healthcare process (29). Third, studies have noted the influence of patients' experience or satisfaction on their willingness to reuse health services (50, 51). Therefore, in this study we integrated the concept of Medical-service Experience into the Anderson model to examine how often the following had occurred in previous healthcare visits: (1) failure to comprehend instructions provided by medical staff, (2) uncertainty about how to pose questions to medical staff, and (3) difficulty reading medication instructions or understanding physicians' advice. Answers were classified as Never (code = 1), Rarely (code = 2), Sometimes (code = 3), Often (code = 4), and Always (code = 5). This categorization enabled a nuanced understanding of the challenges patients face in navigating the healthcare system and their resultant satisfaction levels.

### 2.3 Data analysis

First, we conducted a descriptive analysis of survey participants to examine their demographic characteristics. Next, we conducted exploratory factor analysis (EFA) using SPSS (IBM Corp., Armonk, NY, USA, Version 26) to explore the relationships among the selected variables and ascertain whether the latent variables aligned with the predefined theoretical framework. Specifically, we employed principal component analysis (PCA) to extract item factors and varimax rotation (VR) to enhance the interpretability of the factor solution.

Following this, Structural Equation Modeling (SEM) was implemented in SPSS Amos software, adhering to the two-step approach advocated by Anderson and Gerbing. Within SEM (52), Confirmatory Factor Analysis (CFA) serves as the measurement component, clarifying the associations between latent variables and their respective indicators. The first step was therefore to conduct CFA on each factor to assess the significance of factor loadings. In the second step, grounded in the Andersen Healthcare Utilization Model and subsequent empirical research, we formulated preliminary hypotheses positing that Predisposing Factors, Enabling Resources, Need, Health Behaviors, and Medical-service Experience would predict Decision to Utilize Health Services (DUHS). Furthermore, we hypothesized that Predisposing Factors and Enabling Resources would predict Need and Medical-service Experience; Predisposing Factors would predict Enabling Resources and Health Behaviors; Health Behaviors would predict Need; and Need would predict Medical-service Experience.

In the process of data analysis, we estimated parameters via the maximum-likelihood method. Concurrently, the bootstrap method was used to test latent mediation effects and quantify total, direct, and indirect effects. The model's significance was initially assessed using the χ^2^ value; however, given its sensitivity to large sample sizes, we examined additional goodness-of-fit indices (53). Model fit was evaluated based on the following indices and their respective thresholds for acceptable fit (54, 55): (a) root mean square error of approximation (RMSEA) ≤ 0.08, (b) comparative-fit index (CFI) ≥ 0.90, (c) Tucker–Lewis index (TLI) ≥ 0.90, (d) normed-fit index (NFI) ≥ 0.90, and (e) incremental-fit index (IFI) ≥ 0.90. All statistical tests were conducted at a two-sided significance level of *P* = 0.05.

## 3 Results

### 3.1 Demographic characteristics of participants

A total of 2230 participants were involved in this study. Most were male (55.74%, *n* = 1,243), were married (85.38%, *n* = 1,904), had junior- and senior-high school educations (45.29%, *n* = 1,010), and were enrolled in social medical insurance (80.76%, *n* = 1,801). Percentages of participants living in urban and rural areas were 55.47% (*n* = 1,237) and 44.53% (*n* = 993), respectively. Mean age was 52.39 years (standard deviation [SD], 17.76), and 56.32% (*n* = 1,256) of participants reported an annual income of < 30,000 RMB. These details are summarized in Table 1.

**Table 1:** Demographic characteristics.

**Variables**	**Value**	
Gender	Male	1,243 (55.74)
	Female	987 (44.26)
Age	≤ 30	286 (12.83)
	30–44	472 (21.17)
	45–59	629 (28.21)
	≥60	843 (37.80)
Marital Status	Unmarried	326 (14.62)
	Married	1,904 (85.38)
Education	Primary	765 (34.30)
	Middle	1,010 (45.29)
	Higher	455 (20.40)
Occupation	Unstable employment	1,580 (70.85)
	Self-employed	165 (7.40)
	Employed	485 (21.75)
Income (RMB)	≤ 10,000	863 (38.70)
	10,001–30,000	393 (17.62)
	30,001–50,000	513 (23.00)
	50,001–100,000	349 (15.65)
	≥100,001	112 (5.02)
Medical Insurance	No	122 (5.47)
	Commercial Medical Insurance	20 (0.90)
	Social Medical Insurance	1,801 (80.76)
	Both	287 (12.87)
Region	Urban	1,237 (55.47)
	Rural	993 (44.53)
Total		2,230 (100.00)

### 3.2 Exploratory factor analysis

All variables attained an EFA value of at least 0.856 on the Kaiser–Meyer–Olkin (KMO) test, exceeding the recommended threshold of 0.6. Bartlett's test result was significant (χ^2^ = 17,168.54, *P* < 0.001). Both of these findings demonstrated strong correlations among the variables, indicating that they were suitable for EFA (56).

The initial EFA revealed six factors with eigenvalues exceeding 1. However, MH exhibited substantial loadings (>0.4) on two factors, while Social Support had insignificant loadings (< 0.4) on all factors; therefore, we gradually eliminated these two variables from further analysis. In addition, Gender was grouped with Smoking, Exercise, and Examination, variables that inherently represent dimensions of Health Behaviors, and therefore Gender was removed. After these adjustments, we conducted a second EFA on the remaining 19 variables, resulting in the extraction of five factors through rotation. The cumulative contribution rate of these factors amounted to 61.03%, indicating a substantial representation of total variance. Final EFA results are shown in Table 2.

**Table 2:** The results of the final EFA.

**Factors**	**Items**	**Component**				
		**1**	**2**	**3**	**4**	**5**
Need	Role-physical	0.819				
	Bodily pain	0.807				
	Physical functioning	0.722				
	Social functioning	0.708				
	Role-emotional	0.696				
	General health	0.644				
	Vitality	0.557				
Medical-service experience	Unable to understand medical instructions		0.888			
	Unsure how to ask questions		0.871			
	Unable to read directions/prescriptions		0.819			
Predisposing and enabling	Age			0.774		
	Marriage			0.760		
	Income				0.772	
	Occupation				0.687	
	Education			−0.493	0.512	
	Medical insurance				0.497	
Health behaviors	Physical examination					0.619
	Smoking					0.612
	Exercise					0.588

Based on the above-mentioned EFA results, we grouped Age, Marital, Income, Occupation, Education, and Medical Insurance into the third and fourth factors. However, these groupings did not clearly delineate the two dimensions of Propensity Factors and Enabling Resources as predicted by the Anderson Healthcare Utilization Model. Given that these two dimensions are inherently related to participants' demographic and socioeconomic characteristics and that they showed high intercorrelations, distinguishing them during data analysis proved challenging. Therefore, we merged them into one variable designated Propensity and Enabling. Factors 1, 2, and 5 closely aligned with the pre-established dimensions and were therefore retained as Need, Medical-service Experience, and Health Behaviors, respectively.

Due to the consolidation of Propensity Factors and Enabling Resources, we revised our hypotheses as follows:

**Hypotheses H1–H4:** Predisposing and Enabling, Need, Health Behaviors, and Medical-service Experience would predict DUHS.
**Hypotheses H5–H7:** Predisposing and Enabling would predict Need, Health Behaviors, and Medical-service Experience.
**Hypothesis H8:** Health Behaviors would predict Need.
**Hypothesis H9:** Need would predict Medical-service Experience.

**Figure 2:**
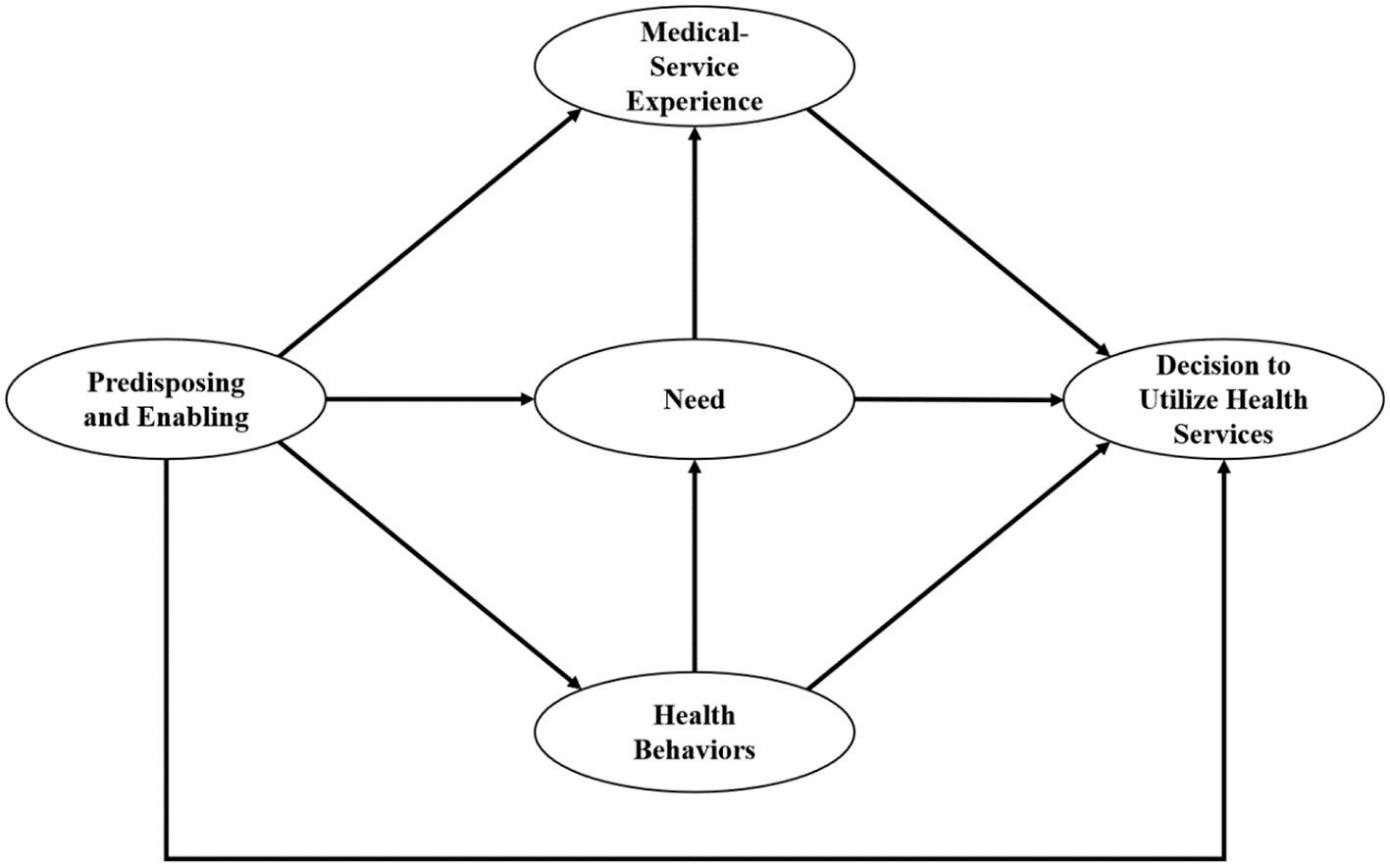
The revised hypothetical structural equation model.

The revised hypothetical model is illustrated in Figure 2.

### 3.3 Confirmatory factor analysis

CFA results indicated that standardized factor loading for Smoking was < 0.1, whereas the remaining variables exhibited high factor loadings; furthermore, all variable factor loadings were statistically significant. Given the emphasis placed on the selected variables in Andersen's model by previous research, in this study we aimed to minimize the number of variables excluded, and consequently we retained all variables in the model. The results are shown in Table 3.

**Table 3:** The results of the CFA.

**Factors**	**Items**	**Factor loading**	**S.E**.	**C.R**.	***P***	**Standardized factor loading**
Need	General health	1				0.627
	Role-physical	1.395	0.044	31.983	< 0.001	0.874
	Bodily pain	1.355	0.044	31	< 0.001	0.821
	Physical functioning	0.675	0.022	30.231	< 0.001	0.788
	Social functioning	0.849	0.036	23.58	< 0.001	0.582
	Role-emotional	0.688	0.03	23.267	< 0.001	0.572
	Vitality	0.693	0.034	20.575	< 0.001	0.484
Medical-service experience	Unable to read directions/prescriptions	1				0.810
	Unable to understand medical instructions	0.952	0.022	42.791	< 0.001	0.853
	Unsure how to ask questions	0.966	0.022	43.487	< 0.001	0.862
Predisposing and enabling	Marriage	1				0.420
	Age	85.155	4.722	18.033	< 0.001	0.711
	Income	−4.134	0.306	−13.528	< 0.001	−0.485
	Occupation	−3.004	0.202	−14.837	< 0.001	−0.538
	Education	−3.791	0.223	−17.015	< 0.001	−0.774
	Medical insurance	−0.902	0.106	−8.47	< 0.001	−0.224
Health behaviors	Frequency of physical examination	1				0.197
	Smoking	0.235	0.076	3.1	0.002	0.086
	Exercise	8.647	2.597	3.33	< 0.001	0.806

C.R., Critical Ratio; S.E., Standard Error; SMC, Squared Multiple Correlation.

### 3.4 Structural model analysis

In SPSS Amos, we computed th*e* β*- a*nd *P*-values of each path to assess the hypothesized relationships. Model results showed that Predisposing and Enabling (β = 0.090; *P* = 0.008) and Medical-service Experience (β = 0.093; *P* < 0.001) had significant positive effects, whereas Need (β = −0.105; *P* < 0.001) had significant negative effects, on DUHS; therefore, H1, H2, and H4 were supported. However, the hypothetical relationship between Health Behaviors and DUHS (β = 0.011; *P* = 0.762) was insignificant, and therefore H3 was not supported. Furthermore, Predisposing and Enabling had a significantly positive effect on both Need (β = 0.463; *P* < 0.001) and Health Behaviors (β = 0.449; *P* < 0.001), confirming H5 and H6. At the same time, this domain had a significant negative effect on Medical-service Experience (β = −0.336; *P* < 0.001), validating H7. Health Behaviors had a significant positive effect on Need (β = 0.134; *P* < 0.001), supporting H8. Finally, Need had a significantly negative effect on Medical-service Experience (β = −0.252; *P* < 0.001), and therefore H9 was supported. Refer to Supplementary Figure S1 for detail.

**Figure 3:**
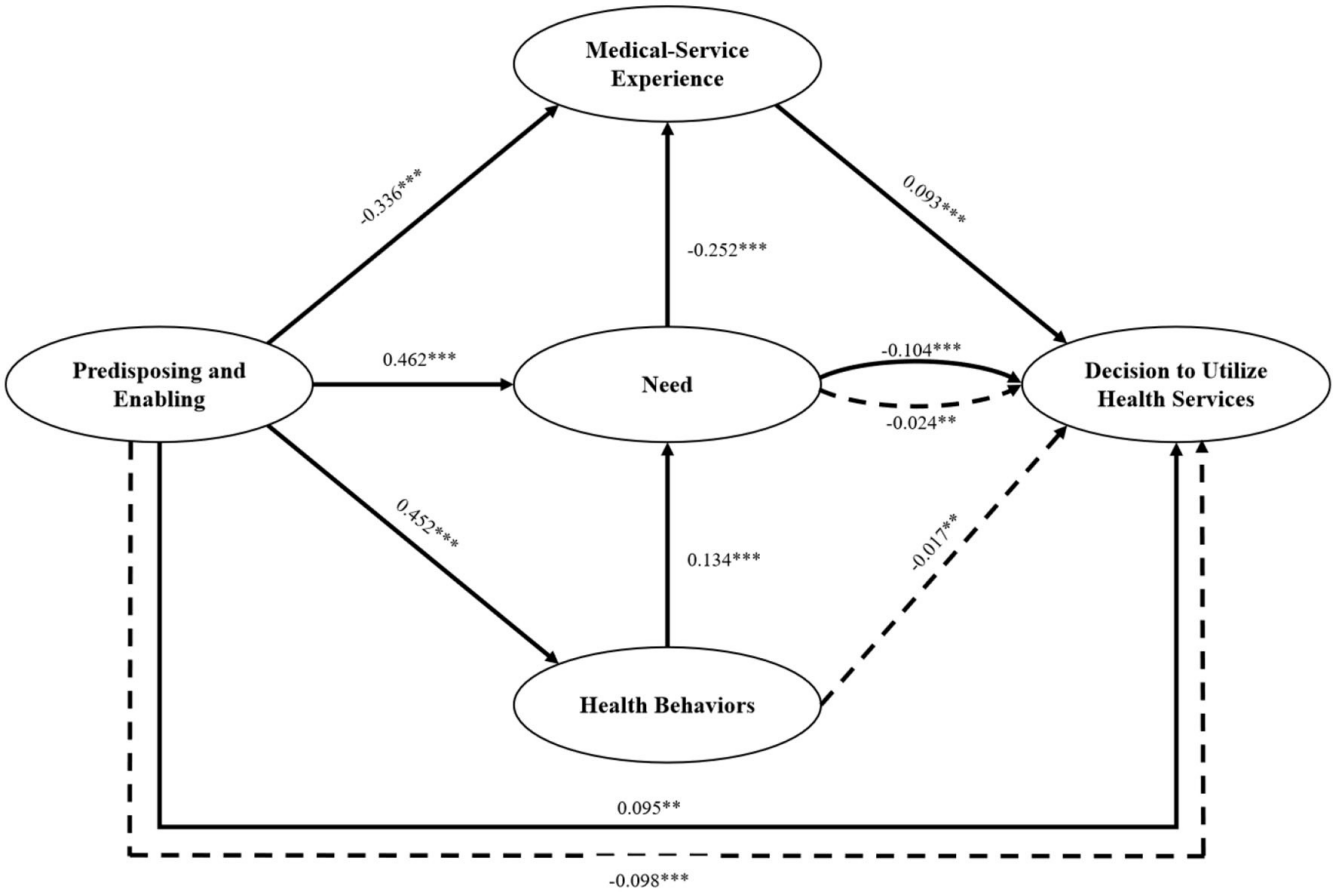
The results of the modified structural equation model. **P < 0.01, ***P < 0.001. Solid lines = direct effect; dashed lines = indirect effect.

After removing the insignificant path, we reassessed the modified model. Besides, on the premise of ensuring the theoretical significance and empirical validity of the resulting model, we employ the modified index to make appropriate adjustments to the model in order to enhance the model's fitting degree. This can increase the model's accuracy and improve its overall performance. The revised SEM results, presented in Figure 3, indicate minimal changes in path coefficients, supporting the assumption of stability. Specifically, the influence coefficients of Predisposing and Enabling, Need, and Medical-service Experience on DUHS were 0.095, −0.104, and 0.093 respectively. The influence coefficients of Predisposing and Enabling on Need, Health Behaviors, and Medical-service Experience were, respectively, 0.462, 0.452, and −0.336. The influence coefficient of Health Behaviors on Need was 0.134, and that of Need on Medical-service Experience was −0.252. Finally, the fit indices of the modified model, presented in Table 4, indicated an acceptable model fit.

**Table 4:** The fit of the structural equation model.

**Model**	**SRMR**	**RMSEA**	**CFI**	**TLI**	**IFI**	**NFI**
Reference	< 0.1	< 0.08	>0.9	>0.9	>0.9	>0.9
Model	0.047	0.058	0.919	0.902	0.920	0.910

### 3.5 Direct- and indirect-effects analysis

We used the bootstrapping function in Amos to scrutinize the model's mediation effect, examining both the direct and indirect effects of each factor on DUHS. Indirect effects were derived from 5,000 bootstrap samples. Table 5 shows the comprehensive breakdown of each factor's direct and indirect effects on DUHS.

**Table 5:** Results of direct, indirect and aggregates of each factor.

**Path**	**Effect**	**Effect sizes**	**Boot SE**	**Z**	***P***	**Bias-corrected; 95%CI**		**Percentile; 95%CI**	
						**Lower**	**Upper**	**Lower**	**Upper**
PE → DUHS	Direct	0.095	0.031	3.065	0.002	0.033	0.154	0.035	0.155
	Indirect^a^	−0.048	0.014	−3.430	0.001	−0.076	−0.022	−0.075	−0.021
	Indirect^b^	−0.006	0.003	−2.000	0.046	−0.014	−0.003	−0.012	−0.002
	Indirect^c^	−0.001	0.001	−1.000	0.317	−0.003	−0.001	−0.003	0.000
	Indirect^d^	−0.031	0.010	−3.100	0.002	−0.051	−0.013	−0.051	−0.013
	Indirect^e^	−0.011	0.004	−2.750	0.006	−0.019	−0.005	−0.018	−0.004
	Total indirect effect	−0.098	0.017	−5.760	< 0.001	−0.132	−0.064	−0.132	−0.064
	Total effect	−0.003	0.023	−0.130	0.896	−0.050	0.042	−0.048	0.043
NE → DUHS	Direct	−0.104	0.029	−3.586	< 0.001	−0.161	−0.048	−0.16	−0.046
	Indirect^f^	−0.024	0.008	−3.000	0.003	−0.040	−0.010	−0.039	−0.009
	Total	−0.127	0.028	−4.536	< 0.001	−0.183	−0.073	−0.181	−0.072
HB → DUHS	Indirect1^g^	−0.014	0.005	−2.800	0.005	−0.025	−0.005	−0.025	−0.004
	Indirect2^h^	−0.003	0.001	−3.000	0.003	−0.007	−0.001	−0.006	−0.001
	Total indirect effect	−0.017	0.006	−2.833	0.005	−0.032	−0.008	−0.030	−0.006
MS → DUHS	Direct	0.093	0.028	3.321	0.001	0.037	0.147	0.039	0.148

^a^PE → NE → RM; ^b^PE → HB → NE → RM; ^c^PE → HB → NE → MS → RM; ^d^PE → MS → RM; ^e^PE → NE → MS → RM; ^f^NE → MS → RM; ^g^HB → NE → RM; ^h^HB → NE → MS → RM. PE, Predisposing and Enabling; NE, Need; HB, Health Behaviors; MS, Medical Service Experience; DUHS, Decision to Utilize Health Services.

The results showed that the direct and indirect effects of Predisposing and Enabling on DUHS were 0.095 and −0.098, respectively. Notably, the largest indirect path coefficient was that of Need, 0.014, which was statistically significant. However, the aggregate effect of Predisposing and Enabling on DUHS was −0.003, which was not statistically significant. Need exerted a direct effect of −0.104 on DUHS, with an additional indirect effect of −0.024 through Medical-service Experience. The aggregate effect was −0.127, which was statistically significant. Health Behaviors demonstrated a purely indirect influence on DUHS, with a statistically significant value of −0.017. Finally, Medical-service Experience had a statistically significant direct effect of 0.093 on DUHS.

## 4 Discussion

Based on Anderson's theoretical framework for healthcare utilization, we meticulously synthesized existing literature and constructed a conceptual model, and carried out a systematic analysis. This model offered a scientific examination of how multidimensional factors—including Predisposing Characteristics, Enabling Resources, Need, Health Behaviors, and Medical-service Experience—individually and collectively contributed to the decision-making process informing residents' non-utilization of healthcare. Consequently, it established a comprehensive mechanism underpinning healthcare utilization behaviors. Rigorous controls were enforced throughout the data collection and processing phases of this research. The findings not only extend the Andersen Healthcare Utilization Model by integrating the medical-service experience, but also reveal the factors that influence residents' health care utilization behavior and the interrelationships among them. This study provides a scientific basis for optimizing medical policies, perfecting the medical service system, and enhancing the overall efficiency of medical utilization.

In this study, we combined the Predisposing Factors and Enabling Resources into a single variable, namely Predisposing and Enabling. During the data analysis, the results of the exploratory factor analysis indicated that these two factors are difficult to distinguish. Besides, we observed that the two dimensions of predisposing factors and enabling resources are intrinsically related to the demographic and socio-economic characteristics of the participants and are highly correlated with each other. From the practical needs of the research and the comprehensive consideration of residents' healthcare utilization behavior, combining these two factors into a single variable can more comprehensively reflect the actual situation of residents in healthcare utilization. It helps us to understand and explain the influencing factors of residents' healthcare utilization behavior more concisely and effectively. According to the results, factor loadings for Income, Occupation, Education, and Medical Insurance were negative, whereas those for Age and Marital Status were positive. The direct influence of Predisposing Characteristics and Enabling Resources on the decision not to utilize healthcare was significant and positive (β = 0.095, *P* < 0.05). This meant that individuals who were older and unmarried and who had lower incomes, less-stable job statuses, lower education levels, and less-comprehensive health insurance coverage exhibited a paradoxical pattern: they had a heightened tendency to require healthcare but were simultaneously more likely to refuse to access it. Research has demonstrated that as individuals age, life and financial stressors tend to intensify, prompting some to adopt cost-saving measures such as delaying seeking medical attention during periods of illness or injury (57). At the same time, human function gradually decreases with age, and older people have cognitive biases about their own states of health (58). On the one hand, they perceive some physical discomfort as a normal phenomenon associated with aging and therefore do not consider medical attention necessary. On the other hand, they may encounter uncomfortable symptoms frequently and attempt to self-diagnose based on their past experiences. Unmarried individuals may face a dearth of direct support and care from partners or family during times of illness or injury (59, 60), resulting in inadequate social support and influencing how they utilize healthcare. Furthermore, compared with married individuals, unmarried people might be more strongly inclined toward independent problem solving and demonstrate less psychological reliance on external support (61). Consequently, they might be more likely to self-manage than to seek medical assistance when dealing with sickness or injury. Conversely, the results showed that individuals with stable jobs, better financial circumstances, and comprehensive health insurance coverage found it easier to access healthcare services without undue concern over financial burdens, similar to previous findings (62–64). Furthermore, those with higher educational attainment tended to have heightened health awareness (65), which facilitated the transformation of healthcare service needs into actual demands. These findings aligned with the results of the existing study. Therefore, health education campaigns and health literacy promotion programme should be targeted at the older adult and unmarried population to reduce “self-diagnosis” behavior. For example, in terms of health education activities, various channels such as community publicity and school education can be used to develop targeted educational content for different age groups and populations In addition, given the relatively low participation rate in commercial insurance, we emphasize the need to provide residents with a diverse range of commercial insurance services, increase participation rates and optimize the reimbursement process, thereby alleviate the economic burden of illness on patients.

Furthermore, our in-depth analysis discovered a nuanced phenomenon: while Enabling Resources made a direct positive contribution to healthcare utilization decisions, these resources unexpectedly exerted a substantial negative indirect influence through mediating factors such as Health Behaviors, Need, and past Medical-service Experience (β = −0.098, *P* < 0.05). This finding implied that when the overall effect of Predisposing and Enabling Resources on healthcare utilization was evaluated, a notable offset between direct and indirect effects appeared, ultimately resulting in a statistically insignificant total effect. To delve deeper into the reason for this phenomenon, we must consider potential interference from external factors. Specifically, the design and execution of policies and systems, entrenched cultural practices, and the operational efficiency of the healthcare system can significantly influence individuals' healthcare utilization decisions (66, 67). These externalities can intricately and imperceptibly interact with Predisposing and Enabling Resources, giving rise to a “masking effect” that obscures or diminishes otherwise discernible direct effects. In light of this, a more scientific and rigorous approach is imperative when formulating and implementing relevant policies and interventions. First, the diversity of population characteristics and variations in Enabling Resources must be fully acknowledged, since they affect healthcare utilization, which will help researchers avoid generalization or simplification. Second, it is crucial to intensify research efforts on individuals with distinct characteristics to comprehend their unique healthcare needs and service utilization patterns and thereby enable the provision of more precise and personalized intervention strategies.

This study theoretically confirms that need is the most influential factor on the utilization of medical services, which is similar to the results of previous researches on Andersen Healthcare Utilization Model (17–19). In this study, we used Health Status as a proxy measure to quantify the demand for medical services among residents. Our findings indicated that Health Status not only exerted a direct positive influence on residents' propensity to seek medical attention when ill or injured but also reinforced this tendency through an indirect pathway involving Medical-service Experience. Specifically, individuals with superior health demonstrated a greater likelihood of seeking medical services, reflecting their favorable stance toward health preservation and acknowledgment of the significance of medical services.

In addition, Health Behaviors exerted a notable indirect influence on healthcare utilization decisions, mediated through Health Status. Proactive engagement in health-promoting behaviors contributes to the preservation and enhancement of an individual's physiological functions, enabling them to maintain or improve their health (68). This favorable state of health not only mitigates the risk of disease and injury but also heightens individuals' awareness and concern for their own wellbeing (69), empowering them to promptly and effectively utilize healthcare when confronted with health challenges. Furthermore, Health Behaviors play a pivotal role in shaping residents' health awareness and preventive attitudes. The consistent practice of healthy behaviors fosters health literacy in residents, heightening their understanding of health knowledge, reinforcing the significance of disease prevention, and motivating them to proactively safeguard their health in daily life (70). This heightened health awareness and preventive mindset serve as a crucial internal stimulus, propelling residents to actively seek medical services when they are ill or injured (71).

Compared with previous researches, this study extends Andersen Healthcare Utilization Model and emphasizes the important impact of “Medical-service Experience” as an independent dimension on health service utilization. In this study, we found that negative encounters with healthcare services markedly deterred patients from seeking care when ill or injured, manifesting as a pattern of “intentional avoidance.” This emphasized that suboptimal healthcare experiences not only fail to address patients' health concerns but can also negatively affect their future utilization of healthcare services over the long term. In the UK, a healthcare quality investigation study reveals that in the patient-provider relationship, poor medical service experiences (such as disrespect or receiving unfair treatment) are highly prevalent, particularly among minority ethnic/racial groups, and this can impact the utilization of healthcare, thereby leading to health disparities (72). Another survey of outpatient department patients indicates that when patients have a poor medical service experience for any reason (such as feeling powerless or being disrespected), their responses may be extremely negative, including disappointment, fear, and the desire to leave the healthcare system (73). A study in the United States shows that a high-perceived quality of healthcare, which is closely related to the medical service experience, will enhance the utilization of healthcare services (74). The research in Bangladesh also demonstrates that the perception of customers plays a crucial role in the utilization of community clinic services (50). For special populations, studies in the United States and India reveal that the negative experiences of the older adult (75) and transgender individuals (76) are important influencing factors of healthcare utilization. Similarly, prior research has emphasized the pivotal role of positive healthcare experiences in shaping favorable healthcare-seeking behaviors (77). Specifically, when residents receive high-quality medical services and experience effective recovery from prior illnesses or injuries, such positive encounters significantly boost their trust in and satisfaction with the healthcare system (77, 78). In addition, Medical-service Experience is an important measure of patient loyalty, its quality intimately tied to patients' subsequent medical-treatment decisions (79). Consequently, in the face of similar health challenges in the future, they draw on their past positive experiences, which make them more inclined to proactively seek professional medical assistance. Therefore, from a practical perspective, in order to foster the effective utilization of healthcare services and enhance patients' health and wellbeing, medical institutions should continuously enhance the quality of medical services, provide patient-centered services to patients, shape a positive patient experience, and promote a long-term and stable doctor-patient relationship (80). In addition, policymakers ought to establish a safer, more efficacious and more convenient setting for patients through enhancing regulatory mechanisms and intensifying personnel training.

### 4.1 Strengths and limitations of this study

Firstly, this study systematically explored the effects of multiple dimensions on residents' healthcare utilization decisions based on the Anderson healthcare utilization model. The multi-dimensional framework offers insights into patients' healthcare-seeking behaviors, providing an empirical basis for health policies and medical service optimization. Secondly, strict controls were imposed in data collection and processing. The CGSS data ensured authority and reliability. Various statistical methods were used to test hypotheses, enhancing the scientific nature and reliability of the results. Ultimately, the study integrates medical service experience into the Andersen model and reveals the interactions among factors. It provides a theoretical foundation for healthcare utilization inquiries and clarifies the decision-making mechanism. The findings offer targeted policy suggestions, which are of great practical significance for improving the healthcare system.

However, some limitations of this research should be acknowledged. First, although many significant factors were considered, potential variables, especially psychological and policy factors, might have been omitted from the analysis. Future research can broaden the variable range, for example, by incorporating factors such as regional disparities, provider trust, and telemedicine access into the research, in order to enhance the explanatory power for residents' healthcare utilization behaviors. Second, this study has limitations in causal inference. To better clarify the causal relationships among various factors, future studies can use more rigorous experimental designs or advanced causal inference methods to enhance the causal explanatory power. Third, there might be potential self-report bias in survey responses. Future research can utilize various data collection methods, such as objective measurement and observation, to mitigate the impact of self-report bias on the research results. Finally, it is essential to highlight that the dataset utilized in this research exclusively originates from China. When extrapolating the findings to international contexts, further verification is indeed necessary, but this study nevertheless offers a significant point of reference for analogous inquiries. It is intrinsically arduous for any scholarly endeavor to achieve exhaustive comprehensiveness, and this investigation is no deviation from this norm.

## 5 Conclusions

In this study, we systematically explored the multi-dimensional factors influencing the healthcare utilization behavior of Chinese residents, effectively validating the applicability and explanatory power of the Anderson Healthcare Utilization Model within this context. Through rigorous empirical analysis, we revealed the intricate interplay among Predisposing Characteristics, Enabling Resources, Health Behaviors, Need, and Medical-service Experience, which collectively molded residents' healthcare-seeking decisions. This study highlights the urgent need for targeted healthcare policies that improve financial accessibility, promote positive healthcare experiences, and support vulnerable groups, particularly older and unmarried individuals. Future research should explore longitudinal trends and intervention efficacy to further refine healthcare access strategies.

## Data Availability

The raw data supporting the conclusions of this article will be made available by the authors, without undue reservation.
