# Supplementary material for: Factors influencing intentional non-utilization of healthcare: a study using the Andersen model
Source: Front Public Health. 2025 Apr 9;13:1503601. doi: 10.3389/fpubh.2025.1503601 (PMC12014544; doi:10.3389/fpubh.2025.1503601)
Supplement: Supplementary file 1 [file Image_1.pdf]

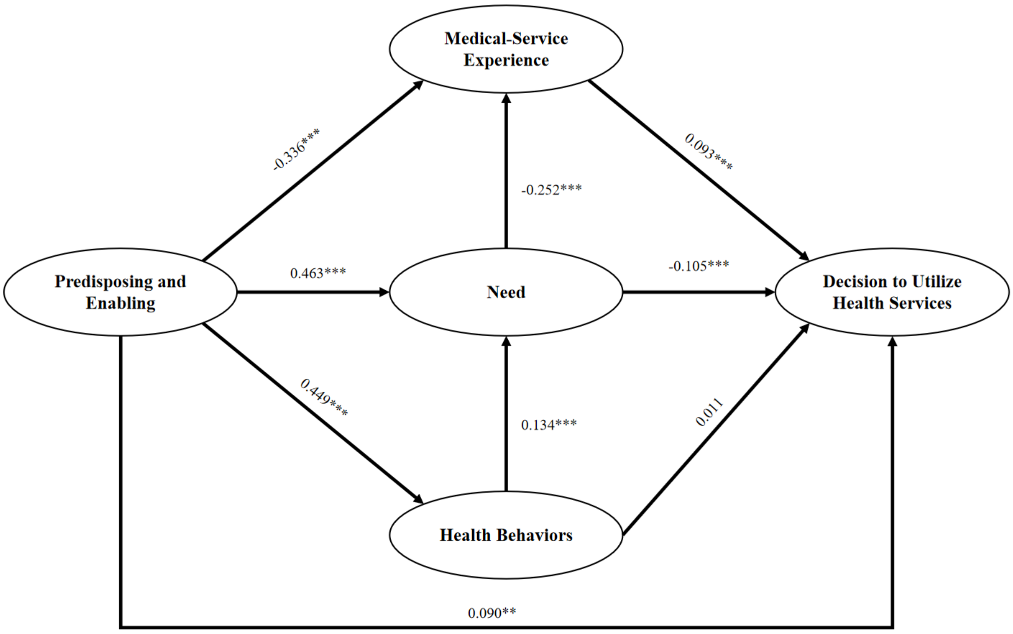

Figure S1. The results of the unmodified structural equation model.

Note: *P < 0.05, ** P < 0.01, *** P < 0.001. Solid lines = direct effect; dashed lines = indirect effect
